# Kirigami–Origami‐Inspired Lead‐Free Piezoelectric Ceramics

**DOI:** 10.1002/advs.202207059

**Published:** 2023-04-25

**Authors:** Zehuan Wang, Denghao Ma, Yunhan Wang, Yan Xie, Zhonghui Yu, Jin Cheng, Li Li, Liang Sun, Shuxiang Dong, Hong Wang

**Affiliations:** ^1^ Department of Materials Science and Engineering Southern University of Science and Technology Shenzhen 518055 P. R. China; ^2^ Institute of Advanced Materials Hubei Normal University Huangshi 435002 P. R. China; ^3^ School of Materials Science and Engineering Peking University Beijing 100871 P. R. China; ^4^ State Key Laboratory for Mechanical Behavior of Materials School of Electronic and Information Engineering Xi'an Jiaotong University Xi'an 710049 P. R. China; ^5^ Institute for Advanced Study Shenzhen University Shenzhen 518051 P. R. China; ^6^ Shenzhen Engineering Research Center for Novel Electronic Information Materials and Devices & Guangdong Provincial Key Laboratory of Functional Oxide Materials and Devices Southern University of Science and Technology Shenzhen 518055 P. R. China

**Keywords:** 3D printing, kirigami, origami, piezoelectric ceramic, sensing

## Abstract

Kirigami‐ and oirigami‐inspired techniques have emerged as effective strategies for material structure design; however, the use of these techniques is usually limited to soft and deformable materials. Piezoelectric ceramics, which are typical functional ceramics, are widely used in electronic and energy devices; however, the processing options for piezoelectric ceramics are limited by their brittleness and feedstock viscosity. Here, a design strategy is proposed for the preparation of lead‐free piezoelectric ceramics inspired by kirigami/origami. This strategy involves direct writing printing and control over the external gravity during the calcination process for the preparation of curved and porous piezoelectric ceramics with specific shapes. The sintered BaTiO_3_ ceramics with curved geometries produced using this strategy exhibit a high piezoelectric constant (*d*_33_ = 275 pC N^−1^), which is 45% higher than that of conventionally sintered sheet ceramics. The curved structure of the ceramics is well‐suited for use in the human body and it was determined that these curved ceramics can detect pulse signals. This strategy can be applied in the large‐scale and low‐cost production of other piezoelectric ceramics with various curved shapes and provides a new approach for the preparation of complex‐shaped ceramics.

## Graphical abstract

A strategy for fabricating kirigami–origami‐inspired lead‐free piezoelectric ceramics is introduced. Extending the technology to the preparation of other ceramics, such as zirconia ceramics, is also feasible. This method can be applied in the large‐scale and low‐cost production of other piezoelectric ceramics with various curved shapes and provides a new approach for the preparation of complex‐shaped ceramics.

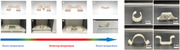

## 1 Introduction

Piezoelectric ceramics, which are typical functional ceramics, are indispensable in various applications, including actuators,^[^ ^1^ ^]^ pressure sensors,^[^ ^2^ ^]^ and piezoelectric energy harvesters,^[^ ^3^ ^]^ owing to their high electromechanical coupling effect, stable mechanical properties, and low cost.^[^ ^4^ ^]^ Although there are advantages of using piezoelectric ceramics for the fabrication of functional devices, processing them into complex geometries is more complex compared to polymers and metals. As most ceramic materials do not contain dislocation deformations, they exhibit high strength but low toughness at low temperatures.^[^ ^5^ ^]^ Conventional piezoelectric ceramics are often sintered into dense materials, and the geometries of the sintered structures are dictated by the shapes of the green bodies. Because the as‐formed materials are fragile and, thus, difficult to treat, the geometries of the resulting piezoelectric ceramics are fixed.

Several attempts have been made to fabricate curved piezoelectric ceramics by direct processing of the sintered ceramic structures, relying on mismatched coefficients of thermal expansion^[^ ^6^ ^]^ or prestressed procedures of the separate layers.^[^ ^7^ ^]^ However, approaches based on coefficients of thermal expansion and mechanically prestressed laminates can only generate simple arc‐shaped laminates with slight curvatures. Additionally, the generality of the laminate structure and the potential for constructing complex 3D or large‐curvature‐containing geometries are limited by the thermal or prestressed post‐processing methods applied. Despite the emergence of 3D ceramic printing processes,^[^ ^8^ ^]^ especially stereolithography technology,^[^ ^9^ ^]^ the fabrication of several 3D ceramic geometries require the use of a support during printing, the removal of which can be time‐consuming. There are fewer reports on the 3D printing of curved ceramics. Furthermore, because the processability of the feedstocks, including the ceramic elements and organic binders, is proportional to the ceramic loading, a compromise always has to be made between these two properties in 3D‐printed ceramics. These competing properties make it difficult to print geometrically complicated ceramic green structures that contain a high volume of ceramic elements and are compact.^[^ ^10^ ^]^

Kirigami and oirigami, ancient Japanese arts of paper folding and cutting, have inspired the development of a variety of scale‐invariant reconfigurable materials containing structures that are arranged spatially or in‐plane.^[^ ^11^ ^]^ These concepts have been endowed with new vitality in the modern times. The use of origami and kirigami techniques have become popular among scientists and engineers because of their predictability, controllability, and scalability in the development of deployable structures,^[^ ^12^ ^]^ reconfigurable metamaterials,^[^ ^13^ ^]^ self‐folding robotics,^[^ ^14^ ^]^ biomedical devices,^[^ ^15^ ^]^ and stretchable electronics.^[^ ^16^ ^]^ Paper and polymers are most commonly used for origami and kirigami as these materials exhibit planar deformation properties.^[^ ^17^ ^]^ Despite their versatility, directly shaping ceramics using origami and kirigami is impractical because of the rigidity and brittleness of conventional ceramics. In contrast, using elastomer‐derived ceramics to produce origami‐shaped ceramic structures will negatively impact the performance of the structure owing to leftover silicon‐based material.^[^ ^18^ ^]^

Herein, we report a strategy for fabricating programmable lead‐free piezoelectric ceramics inspired by kirigami and origami. To create the topology, flat direct writing printing, akin to kirigami, and the effective formation of curved geometries from green bodies through calcination, similar to origami, were used. After polarization, the piezoelectric constant of the sintered piezoelectric ceramic reached 275 pC N^−1^, which is 45% higher than that achieved using similar conventional sheet ceramics. The curved structure conforms to the shape of the human body and can detect pulse signals.

## 2 Results and Discussion

### 2.1 Design of Kirigami‐Inspired Green Bodies

**Figure 1:**
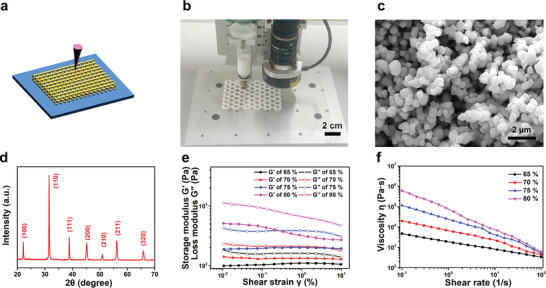
Design of kirigami‐inspired green bodies by 3D printing. a) Schematic showing the direct ink printing of the BaTiO_3_ ceramic (kirigami process). b) Photograph of the direct ink printing worktable. c) Scanning electron microscopy image of the BaTiO_3_ sample. d) X‐ray diffraction pattern of the BaTiO_3_ sample. e) Storage modulus and loss modulus determined by conducting an amplitude sweep of the BaTiO_3_/PVDF inks from 0.01% to 100% strain at room temperature. f) Viscosity of the BaTiO_3_/PVDF inks determined using rotational testing from 0.1 to 100 s^−1^ at room temperature.

**Figure** **1**a depicts the direct ink writing procedure used to conceptualize the green bodies. An optical image of the printing worktable is shown in Figure 1b. The printing parameters are described in Experimental Section and in a literature report.^[^ ^19^ ^]^ The ancient art of paper cutting (kirigami), which transforms planar 2D structures into complex 3D structures.^[^ ^11^ ^]^ Using software design inspired by kirigami, green bodies can be printed in various geometric shapes, including triangles, rectangles, and regular hexagons (Figure S1, Supporting Information). To maintain the integrity and stability of the ceramic green bodies, a higher ceramic weight ratio is vital.^[^ ^20^ ^]^ Figure 1c shows a scanning electron microscopy (SEM) image of the BaTiO_3_ particles prepared using a hydrothermal method. As shown in Figure 1d, the X‐ray diffraction pattern confirms the perovskite crystal structure of BaTiO_3_. Compared with BaTiO_3_ synthesized via the solid phase method, a larger mass fraction of BaTiO_3_ produced using the hydrothermal method could be mixed into the polyvinylidene fluoride (PVDF)/N, N‐dimethylformamide (DMF) solution. This is owing to the formation of a hydrogen bond between the hydroxyl group on the surface of BaTiO_3_ and the fluorine atom on the PVDF molecular chain,^[^ ^21^ ^]^ improving the compatibility and dispersibility of BaTiO_3_ in the mixture.^[^ ^22^ ^]^ To evaluate the dispersion degree of the hydrothermally produced BaTiO_3_ ceramic particles, the surface potentials of the particles produced using the two methods were investigated (Figure S2, Supporting Information). BaTiO_3_ produced by the hydrothermal method is pH sensitive and easily protonates in water; thus, the hydroxyl group on the particle surface is Brønsted basic.

BaTiO_3_‐PVDF gel containing a ceramic weight percent between 65 wt.% and 80 wt.% was extruded through the printer nozzle. Figure 1e shows the amplitude sweep results for the different weight percentages of BaTiO_3_‐PVDF gels. All the BaTiO_3_‐PVDF gels display dominant storage moduli over their loss moduli, suggesting that they exhibit gel‐printable features. Figure 1f shows the viscoelasticity of the BaTiO_3_‐PVDF gels. All the BaTiO_3_‐PVDF gels exhibit shear thinning behavior which is associated with viscoelastic solids; this indicates that they are suitable for 3D printing via extrusion. However, an increase in the BaTiO_3_ weight percentage to 83 wt.% significantly increases the viscosity of the gel (Figure S3, Supporting Information). Therefore, the 80 wt.% BaTiO_3_‐PVDF gel was selected for the direct ink printing process. The green bodies were printed in various geometric shapes and analyzed by thermogravimetric analysis (TGA) to evaluate the calcination process (Figure S4, Supporting Information). The initial 1.6 wt.% weight loss observed is due to solvent evaporation. An increase in the temperature to 450 °C causes PVDF, which acts as a caking agent, to be volatilized. The calcination temperature profile is shown in Figure S5 (Supporting Information).

### 2.2 Origami Preparation Strategy

**Figure 2:**
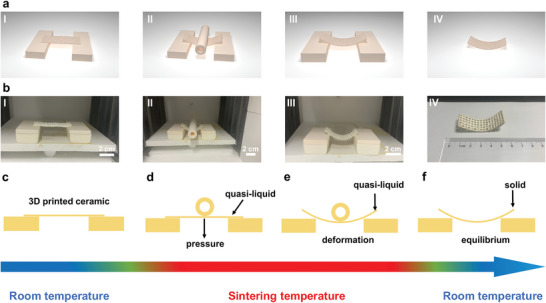
Preparation of oirigami‐inspired ceramics process and setup for the ceramic synthesis. a) Images representing the origami process. An alumina tube is placed on the green body prior to calcination. The force equilibrium of the initial stage is disrupted during the deformation stage owing to the quasi‐liquid state of the sintering specimen at high temperatures. In the equilibrium step, a new force equilibrium is established, and the curved configuration persists until the final stage. b) Optical images showing the formation of the curved geometry. These images validated the origami method. c–f) Simplified models representing the origami process.

A schematic diagram of the manufacturing process for the origami‐inspired curved porous BaTiO_3_ ceramics is shown in **Figure** **2**a. The porous BaTiO_3_ green body was mounted on two alumina platforms that have low thermal expansion and good thermal conductivity; this is advantageous for constructing a stable high‐temperature system in a furnace. A hollow alumina tube was placed in the center of the BaTiO_3_ green body prior to calcination, and as the temperature was increased, the sintered body transitioned from a solid to a quasi‐liquid state. During the calcination process, the gravity of the hollow alumina tube dominates the deformation process. The force balance established in the original solid‐state material is disrupted owing to the viscous properties of the quasi‐liquid sintered material. Porous BaTiO_3_ ceramics are also deformed during calcination. As the deformation continues, a new equilibrium is produced, and gravity is eventually balanced by a change in the internal shear forces.^[^ ^23^ ^]^ Finally, ceramic sintered solids with curved geometries are created because the system is thermally and mechanically stable.

Figure 2b illustrates the ceramic origami process; the progression of the sintered body configuration was recorded. Photographs I–IV show how the curved geometry was obtained. Sectional perspectives of the origami process are presented in Figure 2c–f. Because the alumina tube does not deform during sintering and it has a high thermal conductivity, the underlying BaTiO_3_ green body will bend with an increase in the temperature. This results in a significantly curved structure being formed during the heating and early sintering stages of the process. The geometry of the material is governed by the curvature of the final sintered body. Because a sintered body is more malleable than a ceramic sintered body, it is ideal for shape modeling.^[^ ^24^ ^]^

### 2.3 Characterization and Performance of the Kirigami–Origami Ceramics

**Figure 3:**
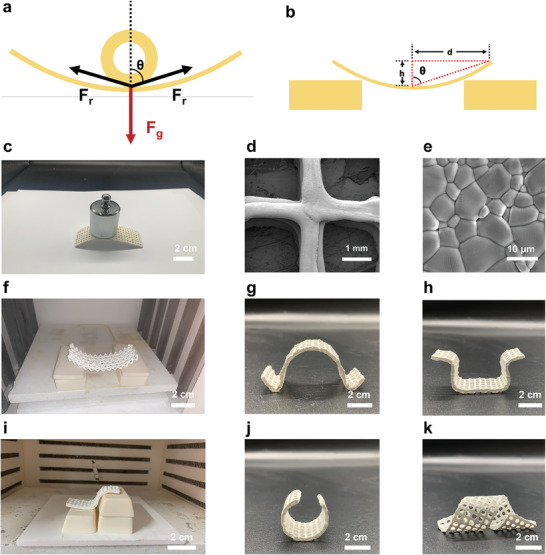
Tunability study of the kirigami–origami ceramic process and samples presentation. a, b) Force analysis of the simplified models in a load balance state. c) Photograph showing the kirigami–origami lead‐free piezoelectric ceramic bearing a load of 500 g. d) SEM image of the calcined kirigami–origami BaTiO_3_ ceramic. e) SEM image of the curved surface of the obtained BaTiO_3_ ceramic. f) Optical image of the calcined kirigami–origami ZrO_2_ ceramic. g–k) Photographs of the kirigami–origami BaTiO_3_ ceramics with different shapes.

To investigate the tunability of the kirigami–origami ceramic process, we fabricated curved BaTiO_3_ ceramics under mechanical boundary conditions. A simplified model, in which gravity, *F*_g_, is balanced by an equivalent resistance, *F*_r_, was created, as illustrated in **Figure** **3**a. A substantial resistance is required to construct a new equilibrium in the configuration after breaking the initial equilibrium; this primarily relies on the angle, *θ*, and the weight of the alumina tube (Figure 3b). We also sintered large‐curvature ceramics by designing corresponding mechanical boundary conditions and adjusting the weight of the alumina tube (Figure S6, Supporting Information). The resulting sintered ceramic has the same curvature as the inner surface of the supporting tube (Figure S7, Supporting Information). To demonstrate the generality and customizability of the kirigami–origami process, we implemented our strategy using a range of different conditions. The kirigami–origami fabrication process allows for the simultaneous sintering of several samples, which is extremely advantageous when considering upscaling the production of these piezoelectric ceramics.

As shown in Figure 3c, the curved porous BaTiO_3_ ceramics could withstand a load of 500 g, suggesting that the design is mechanically strong. Figure 3d shows a SEM image of the calcined kirigami–origami BaTiO_3_ ceramic. After de‐binding and sintering the BaTiO_3_ ceramics, a dense material is obtained (Figure 3e). The final density of the sintered BaTiO_3_ specimen was 5.79 g cm^−3^; its relative density exceeded 96% of the theoretical value (theoretical density: 6.02 g cm^−3^). To evaluate and validate the kirigami–origami ceramic fabrication strategy, curved porous zirconia ceramics were fabricated (Figure 3f). The density of the sintered ZrO_2_ ceramic was 5.85 g cm^−3^, which is 96.7% of the theoretical value (6.05 g cm^−3^). This indicates that the kirigami–origami ceramic fabrication method is highly beneficial for the formation of high‐density materials. Curved porous ceramics with various shapes can be created by repositioning the aluminum oxide tubes or cubes. Figure 3g–k and Figure S8 (Supporting Information)shows kirigami–origami BaTiO_3_ ceramics with different shapes. Much more images of different angles of samples was presented in Figure S9 (Supporting Information).

**Figure 4:**
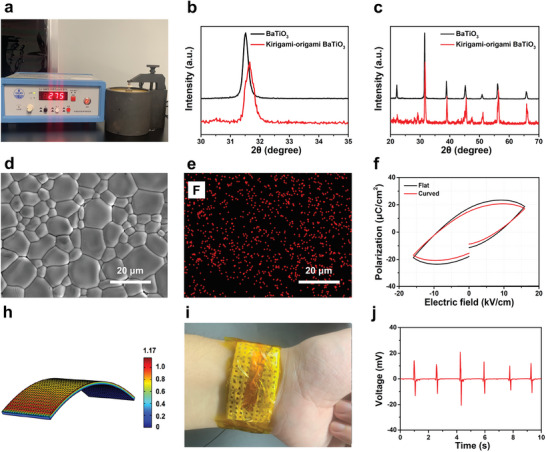
Structure analysis of the kirigami–origami BaTiO_3_ ceramic and piezoelectric sensor application. a) The piezoelectric coefficient *d*_33_ of the polarized kirigami–origami BaTiO_3_ ceramic. b) X‐ray diffraction pattern of the calcined kirigami–origami BaTiO_3_ ceramic. c) Magnified section of the X‐ray diffraction patterns of the BaTiO_3_ samples. d) SEM image of the surface of the kirigami–origami BaTiO_3_ ceramic. e) Energy‐dispersive spectroscopy (EDS) maps of the surface of the kirigami–origami BaTiO_3_ ceramic. f) A comparison between the polarization‐electric field loops obtained at 10 Hz of the kirigami–origami BaTiO_3_ and conventional sintered BaTiO_3_ ceramics. g) A color‐coded simulation of the polarized kirigami–origami BaTiO_3_ ceramic. h) Optical image of the kirigami–origami BaTiO_3_ ceramic applied as a pulse testing sensor. i) The output voltages of the kirigami–origami BaTiO_3_ ceramic applied as a sensor.

**Figure** **4**a shows the piezoelectric constant test conducted on the kirigami–origami BaTiO_3_ ceramics. The piezoelectric constant of the sintered piezoelectric ceramics reaches 275 pC N^−1^ after polarization, which is 45% higher than that of typical sintered BaTiO_3_ ceramics.^[^ ^25^ ^]^ Measuring samples of sintered BaTiO_3_ ceramics with different geometry and pore size, the piezoelectric constant was not changed. The sintered kirigami–origami BaTiO_3_ ceramics were also analyzed using X‐ray diffraction; the X‐ray diffraction pattern of the kirigami–origami BaTiO_3_ ceramics is shifted compared to that of conventional BaTiO_3_ (Figure 4b). As illustrated in Figure 4c, the magnified section of the X‐ray diffraction pattern clearly indicates a shift in the (110) phase of the material, suggesting that the BaTiO_3_ lattice expanded with the introduction of foreign atoms. The variation in lattice parameters of samples was also changed (Table S1, Supporting Information). Figure 4d,e display the energy‐dispersive spectroscopy (EDS) maps of the surface of the kirigami–origami BaTiO_3_ ceramic. Fluorine is detected in the kirigami–origami BaTiO_3_ ceramic. PVDF is the only compound used in the fabrication of the BaTiO_3_ ceramic which contains fluorine. Figure 4f shows the ferroelectric polarization versus electric field (*P*–*E*) loops of the kirigami–origami BaTiO_3_ and conventional sintered BaTiO_3_ ceramics obtained at ambient temperature and 10 Hz; the results for the two BaTiO_3_ ceramic samples are similar. COMSOL Multiphysics, along with the finite element method, was used to investigate the output voltage of the kirigami–origami BaTiO_3_ ceramic, as shown in Figure 4g. The simulated results indicate that under an applied stress of 20 N, the BaTiO_3_ ceramic can generate ≈1.17 V. The curve of the prepared sample is ergonomic (Figure S10, Supporting Information). By combining the polarized kirigami–origami BaTiO_3_ ceramic with a polyimide film and conducting wires a curved sensor could be fabricated. Due to the protection of polyimide film, the device will not harm to users(Figure S11, Supporting Information). This sensor can be easily attached to the surface of the skin at the neck, shoulders, elbows, and wrists without resulting in any discomfort (Figure 4h). To further verify the feasibility of the sensor, the piezoelectric signal from the sensor was collected using an oscilloscope. Figure 4i shows the output voltage of the pulse signal from the polarized kirigami–origami BaTiO_3_ ceramic sensor. Based on the feasibility experiment, kirigami–origami BaTiO_3_ ceramics can potentially be applied as self‐sensing materials and wearables.

## 3 Conclusion

In this paper, we introduced a strategy for fabricating kirigami–origami‐inspired lead‐free piezoelectric ceramics. We proposed a general effective method for the design of a kirigami‐inspired ceramic structure and softening of the rigid ceramic through calcination for origami‐inspired shape forming. Our kirigami–origami ceramics exhibited designable configurations and high compactness. Extending our technology to the preparation of other ceramics, such as zirconia ceramics, is also feasible; this method is a practical approach for the design of ceramics with complex structures. In addition, owing to fluorine doping, the kirigami–origami BaTiO_3_ ceramic prepared has a higher piezoelectric constant than conventional ceramics. A sensor created using the polarized kirigami–origami BaTiO_3_ ceramic can detect pulse signals, which promotes the application of these ceramic materials in the development of wearable electronics and health monitoring devices.

## 4 Experimental Section

### Inks Preparation

BaTiO_3_ particles were synthesized for the 3D printing inks using a hydrothermal method. Commercial PVDF powder and DMF were used to bind the BaTiO_3_ powder to manufacture ceramic suspensions. To prepare extrudable ceramic suspensions with a high content of BaTiO_3_ powder, PVDF was first dissolved in DMF at a weight ratio of 1:9. BaTiO_3_/PVDF inks containing different weight percentages of BaTiO_3_ nanoparticles were formulated. The BaTiO_3_/PVDF inks, which were mixed by stirring with a glass rod, were transferred to the printing syringes.

### Sample Preparation

3D computational design software was used to support the printheads. The printheads were connected to a set of ink‐filled syringes mounted on a three‐axis linear‐motion controller with air bearings. Digital pressure regulators were used to supply pressure to the syringes. The inks were printed on a polyethylene glycol terephthalate (PET) film using a nozzle with a diameter of 260 or 410 µm. A constant printing speed of 160 mm min^−1^ and extrusion pressure of 0.8–1.0 MPa were used. The printed samples were dried at 80 °C for 6 h to avoid the formation of microcracks. Alumina tubes were placed on the dried green samples, which were then sintered in a muffle furnace. The samples were heated to 600 °C using a ramp rate of 3 °C min^−1^ and holding time of 2 h to allow the binder to be vaporized. Subsequently, the green bodies were sintered at 1320 °C for 2 h and cooled to room temperature.

### Parameter Measurement

Microscopy and compositional analyses were conducted through SEM (TESCAN); the microscope was operated at 10 kV. An X‐ray diffractometer with CuK*α* radiation (D8 Advance, Bruker) was used to obtain the X‐ray diffraction patterns of the samples. The zeta potentials of the BaTiO_3_ samples were determined in aqueous solutions at different pH values using a Zetasizer Nano ZS90 (Malvern) operated at room temperature. The thermal stabilities of the samples were characterized by TGA. TGA was conducted on a TG209F3 instrument (NETZSCH Scientific Instruments) by ramping the temperature from room temperature to 1000 °C at a heating rate of 10 °C min^−1^ under a flow of air. The elemental compositions and chemical states of the samples were verified using an X‐ray photoelectron spectrometer (Axis Ultra, Kratos Analytical, Ltd.) operated at an X‐ray power of 150 W. Monochromatic Al K*α* (h*ν* = 1486.7 eV) radiation was used as the excitation source. All the spectra were calibrated using the C 1s peak (284.7 eV). The polarization‐electric field hysteresis (*P*–*E*) loops of the samples were obtained using a Precision Multiferroic II instrument (RADIANT Technologies inc.). Rheological measurements were performed on a rheometer (HAAKE MARS III). A quasi‐static piezoelectric meter (ZJ‐3AN, Institute of Acoustics, Beijing, China) was used to evaluate the piezoelectric performances of the samples. The output voltages were measured using a digital storage oscilloscope (KEYSIGHT MSOX4024A).

## Conflict of Interest

The authors declare no conflict of interest.

## Supporting information

Supporting Information

## Data Availability

The data that support the findings of this study are available from the corresponding author upon reasonable request.
