# Supplementary material for: Kirigami–Origami‐Inspired Lead‐Free Piezoelectric Ceramics
Source: Adv Sci (Weinh). 2023 Apr 25;10(17):2207059. doi: 10.1002/advs.202207059 (PMC10265079; doi:10.1002/advs.202207059)
Supplement: Supplementary file 1 — Supporting Information [file ADVS-10-2207059-s001.pdf]

ADVANCED SCIENCE
Open Access

Supporting Information

for *Adv. Sci.*, DOI 10.1002/advs.202207059

Kirigami–Origami-Inspired Lead-Free Piezoelectric Ceramics

Zehuan Wang, Denghao Ma, Yunhan Wang, Yan Xie, Zhonghui Yu, Jin Cheng, Li Li, Liang Sun, Shuxiang Dong and Hong Wang**

Supporting information

Kirigami-origami-inspired lead-free piezoelectric ceramics

Zehuan Wang, Denghao Ma, Yunhan Wang, Yan Xie, Zhonghui Yu, Jin Cheng, Li Li, Liang Sun, Shuxiang Dong, Hong Wang *
Z. Wang, J. D. Ma, Cheng, Y. Wang, Y. Xie, J. Cheng, *L. Li, L. Sun*, H. Wang
Department of Materials Science and Engineering,
Southern University of Science and Technology,
Shenzhen 518055, China
E-mail: wangh6@sustech.edu.cn
Z. Wang
Institute of Advanced Materials,
Hubei Normal University,
Huangshi 435002, China
E-mail: zehuanwang@hbnu.edu.cn
Z. Wang, Z. Yu, S. Dong
School of Materials Science and Engineering
Peking University
Beijing 100871, China
H. Wang
Shenzhen Engineering Research Center for Novel Electronic Information Materials and Devices & Guangdong Provincial Key Laboratory of Functional Oxide Materials and Devices
Southern University of Science and Technology,
Shenzhen 518055, China
S. Dong
Institute for Advanced Study
Shenzhen University
Shenzhen 518051, China
D. Ma,
State Key Laboratory for Mechanical Behavior of Materials
School of Electronic and Information Engineering
Xi'an Jiaotong University
Xi'an 710049, China

Keywords: origami, kirigami, 3D printing, piezoelectric ceramic, sensing

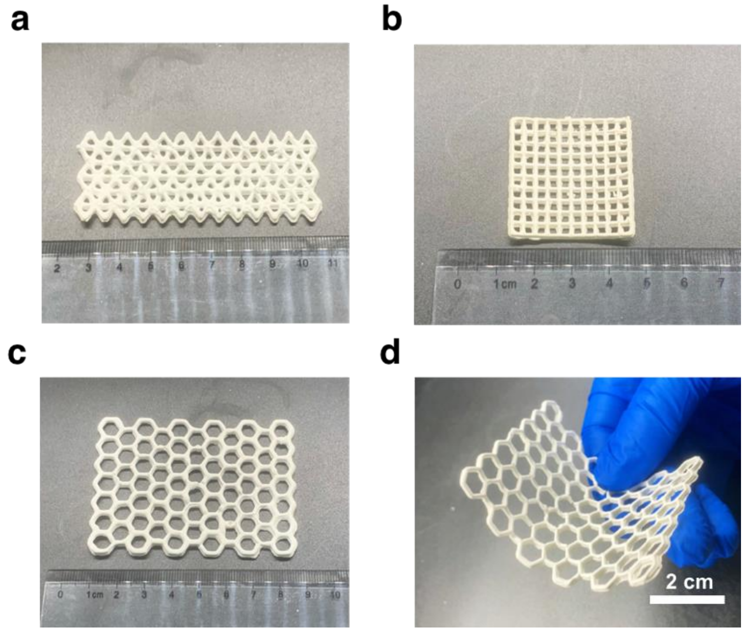

Figure S1 the geometry shapes of green bodies. (a) Triangle. (b) Rectangle. (c, d) Regular hexagon.

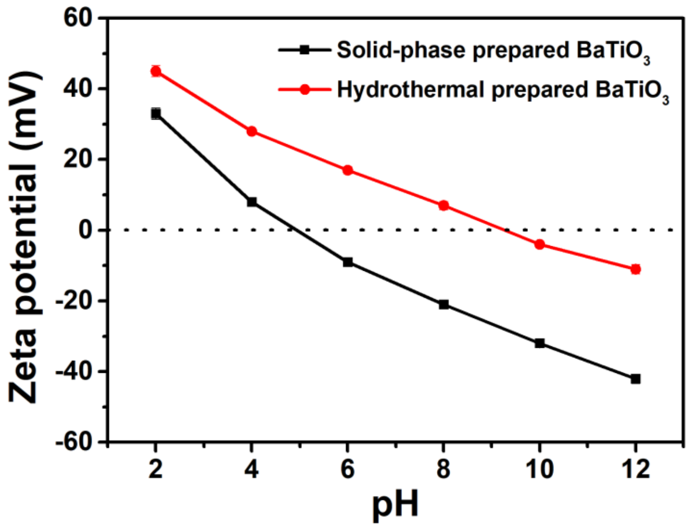

Figure S2 Zeta-potential of $BaTiO_3$ particles dispersed in aqueous solution with different pH values.

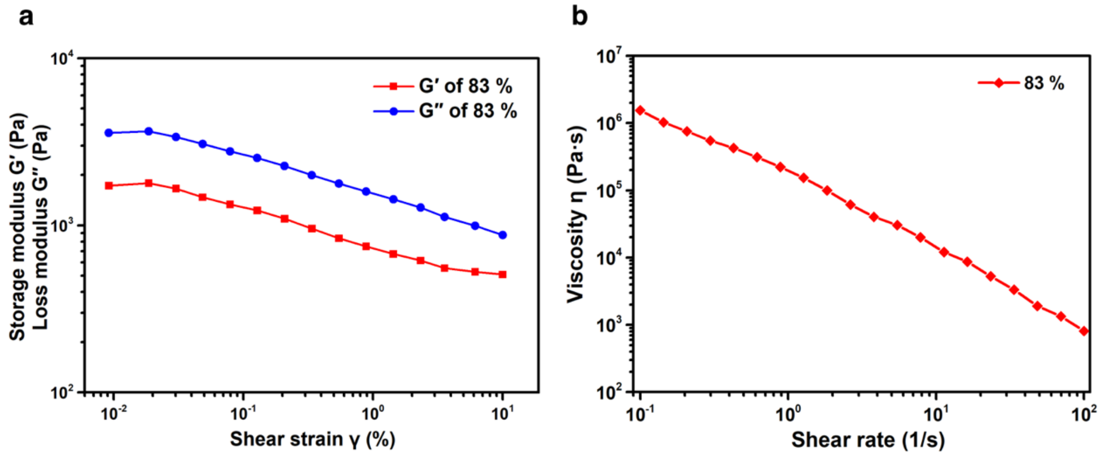

Figure S3 (a) Measured storage modulus and loss modulus from amplitude sweep of 83wt.% $BaTiO_3$/PVDF inks system from 0.01% to 100% strain at room temperature. (b) Measured viscosity of 83wt.% $BaTiO_3$/PVDF inks system from rotational testing from 0.1 s^{-1} to 100 s^{-1} at room temperature.

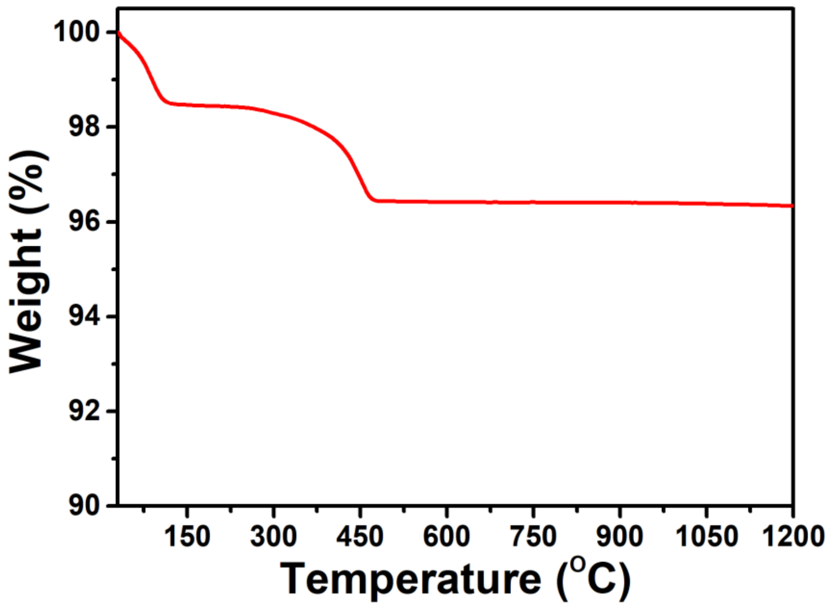

Figure S4 Thermogravimetric Analysis curves of 3D printed $BaTiO_3$ green body.

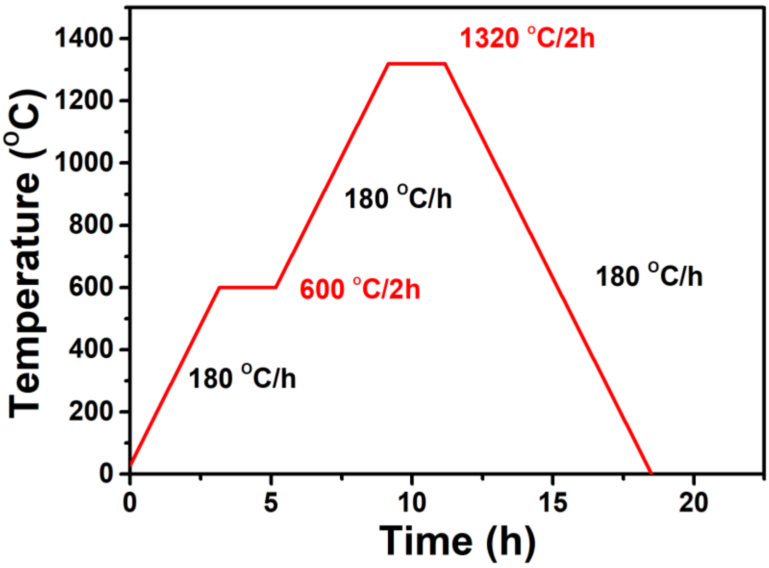

Figure S5 Temperature profile of green body calcination process.

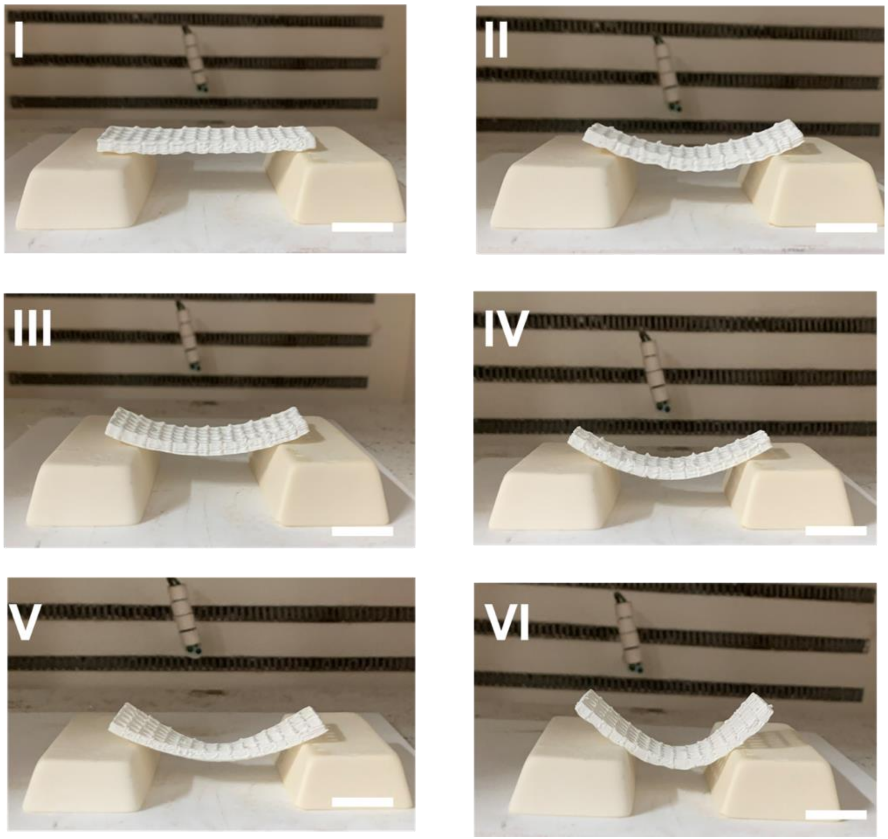

Figure S6 Photographs of the sintered $BaTiO_3$ ceramics by loading different weight of alumina tube. Scale bar is 2 cm.

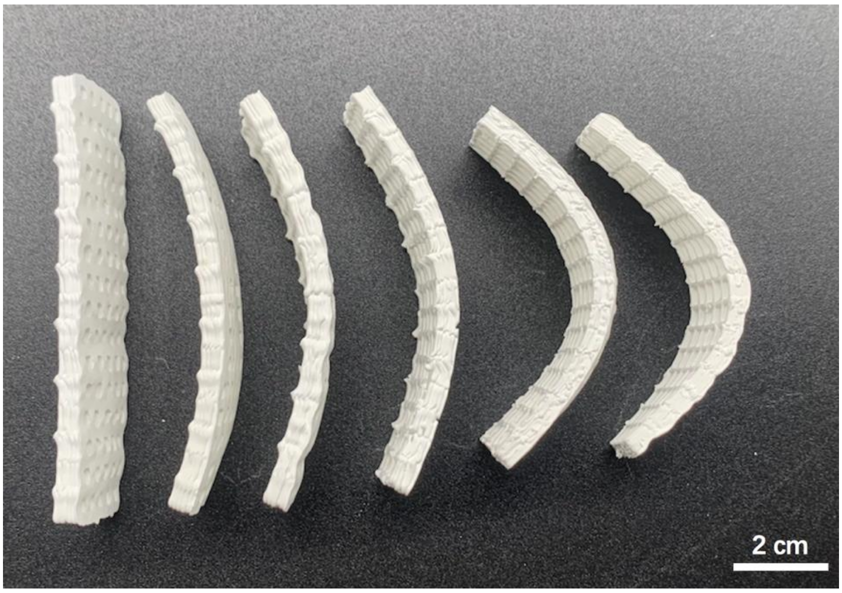

Figure S7 Top view of the sintered $BaTiO_3$ ceramics shown in Figure S6 (from I to VI).

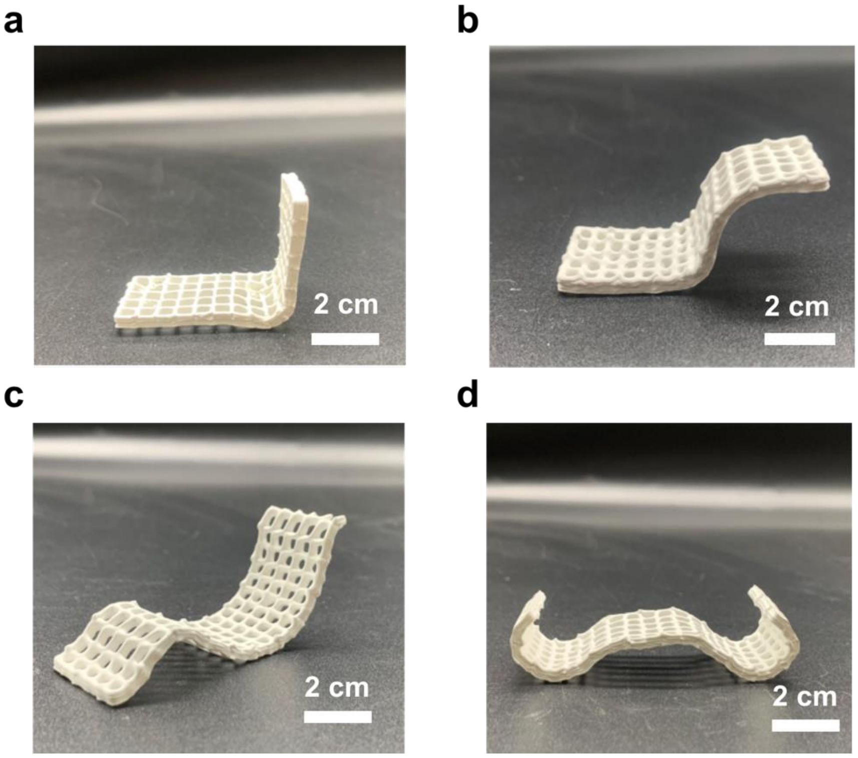

Figure S8 Photographs of the kirigami-oirigami $BaTiO_3$ ceramic with different shapes.

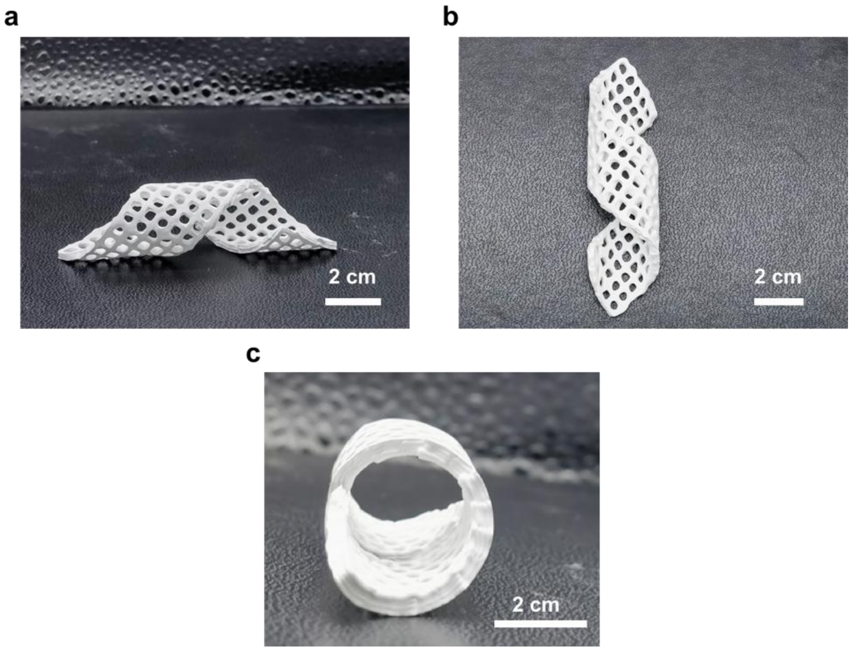

Figure S9 Photographs of samples with different angles .

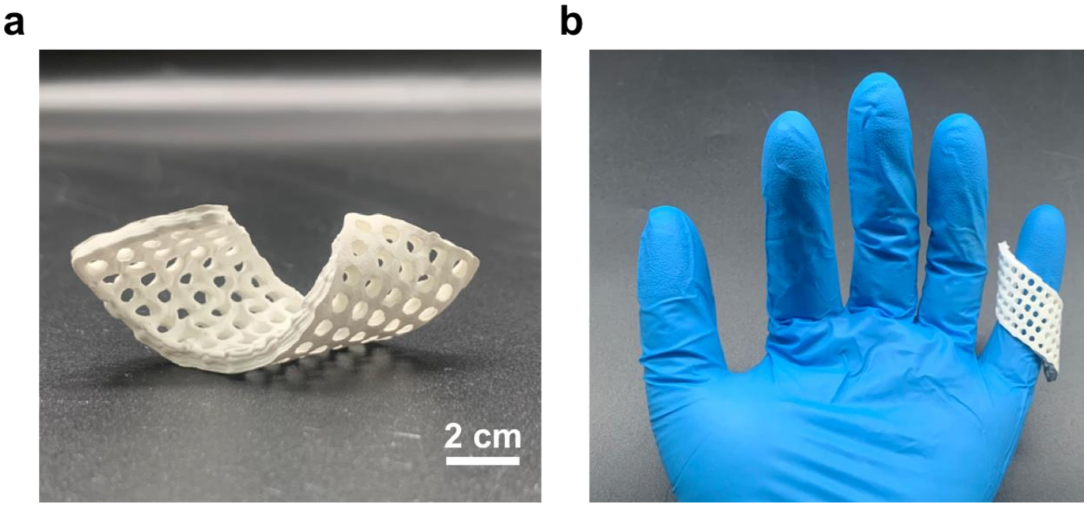

Figure S10 (a) Photographs of the kirigami-oirigami $BaTiO_3$ ceramic. (b) Sample could be a ring on the finger.

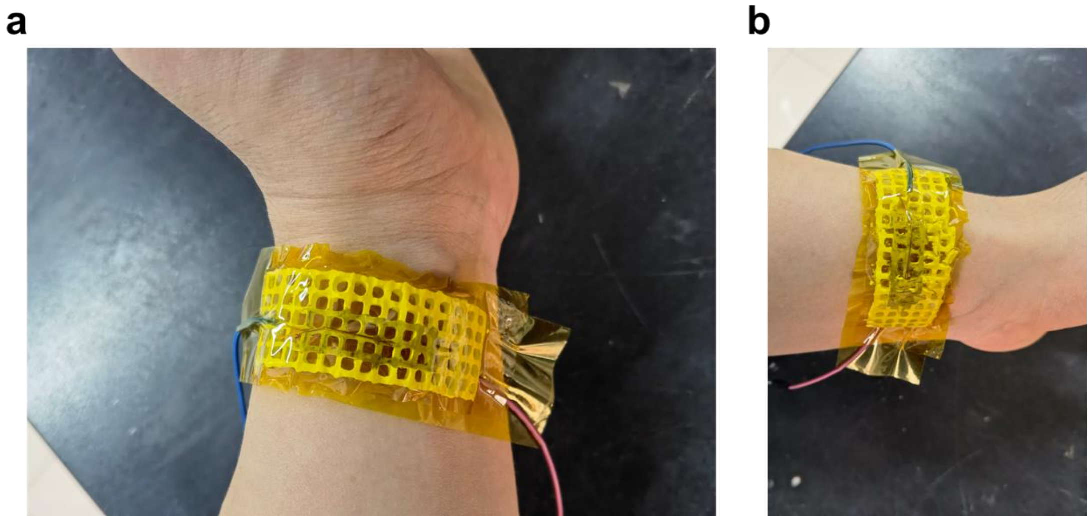

Figure S11 Images of (a)intact and (b)cracked devices.

Table S1 Lattice parameters with a, b and c of the kirigami-origami $BaTiO_3$ ceramics and conventional $BaTiO_3$ ceramics.

Samples	a	b	c
Kirigami-origami $BaTiO_3$	3.9952	3.9952	4.0319
Conventional $BaTiO_3$	3.9948	3.9948	4.0342
